# Effects of Adipose-Derived Mesenchymal Stem Cell-Secretome on Pyroptosis of Laparoscopic Hepatic Ischemia Reperfusion Injury in a Porcine Model

**DOI:** 10.3390/cells14100722

**Published:** 2025-05-15

**Authors:** Yajun Ma, Lei Cao, Pujun Li, Zhihui Jiao, Xiaoning Liu, Xiangyu Lu, Tao Liu, Hongbin Wang

**Affiliations:** 1 College of Veterinary Medicine, Northeast Agricultural University, Harbin 150030, China; mayajun1994@126.com (Y.M.); caolei_001010@163.com (L.C.); pujunli1024@163.com (P.L.); liuxiaoningneau@163.com (X.L.); lxy1997202206@163.com (X.L.); liutaotiger@163.com (T.L.); 2 College of Wildlife and Protected Area, Northeast Forestry University, Harbin 150030, China; zhihuijiao@nefu.edu.cn

**Keywords:** paracrine effects, ADSC-secretome, minipig, HIRI, pyroptosis

## Abstract

Extensive research has been conducted on mesenchymal stem cells (MSCs) regarding their ability to modify the immune response and reduce tissue damage. Many researchers have found that the regulatory capacity of MSCs primarily comes from their secretome. As a result, there has been much interest in utilizing “cell-free” therapies as alternatives to stem cell treatments. In this study, the secretome from adipose mesenchymal stem cells (ADSC-secretome) was extracted and injected into minipigs with established liver injury models. Blood and liver tissue samples were obtained prior to the procedure, as well as on days 1, 3, and 7 after surgery. It was found that ADSC-secretome effectively suppressed the synthesis of the NOD-like receptor protein 3 (NLRP3) inflammasome, leading to a downregulation of gasdermin-D (GSDMD) expression, and demonstrated a more prominent anti-pyroptosis effect compared to ADSCs. Furthermore, ADSC-secretome inhibited the high mobility group box 1 (HMGB1)/toll-like receptor 4 (TLR4)/nuclear factor kappa-B (NF-κB) inflammatory pathway. In summary, both ADSC-secretome and ADSCs inhibited pyroptosis in right hemihepatic ischemia–reperfusion combined with left hemihepatectomy injury, and ADSC-secretome exhibited a stronger therapeutic effect. ADSC-secretome exerted these therapeutic effects through the inhibition of the HMGB1/TLR4/NF-κB inflammatory pathway. In the future, “cell-free” therapy is expected to replace cell-based methods.

## 1. Introduction

Hepatic ischemia–reperfusion injury (HIRI) occurs when blood and oxygen supply to the liver is temporarily and selectively impeded, followed by reperfusion [1]. At the molecular level, HIRI is associated with overproduction of reactive oxygen species (ROS), resulting in cellular death through peroxidation, along with the release of cytokines and chemokines that contribute to the inflammatory process. The stimulation of immune cells and the generation of adhesion factors promote massive infiltration of neutrophils, which eventually damages the microvascular system and induces endothelial cell dysfunction, thereby exacerbating tissue damage [2,3]. HIRI not only destroys the liver structure and impairs liver function and metabolism [4], but can also cause injuries to the lungs [5], brain [6], and kidneys [7].

Pyroptosis involves inflammation and is a caspase1-dependent process of programmed cell death [8]. During the onset of HIRI, low oxygen levels encourage the activation and buildup of neutrophils and macrophages in the liver, worsening tissue damage by releasing ROS and inflammatory cytokines through paracrine and autocrine signaling [9]. Severe inflammatory response is the main influence of HIRI [10]. Furthermore, pyroptosis predominantly occurs in innate immune cells rather than in hepatocytes [11,12]. In a murine model of HIRI, inhibiting NLRP3 and caspase1 led to a prevention of NLRP3 inflammasome activation, which in turn reduced liver damage [13]. Li et al. additionally demonstrated that specifically knocking down GSDMD in Kupffer cells mitigated HIRI [14]. In fact, pyroptosis is implicated in the pathophysiology of various forms of IRI [15].

Multiple inflammatory factors can induce the release of HMGB1, mediating inflammatory responses. In the early stages of HIRI, HMGB1 regulates inflammation and organ damage [16,17]. It binds to and activates TLR4, a receptor responsible for recognizing patterns that orchestrates the inflammatory response associated with HIRI [18] through the NF-κB signaling cascade [19]. As a principal regulator of inflammation and immune balance, NF-κB represents a significant target for new anti-inflammatory therapies [20]. When NF-κB is activated, it promotes the transcription of genes that code for proteins related to the NLRP3 inflammasome [21]. This activation process ultimately results in the augmented production and release of key inflammatory cytokines, such as IL-1β, TNF-α, and IL-6, which play critical roles in mediating the body’s inflammatory responses. The pathway involving HMGB1/TLR4/NF-κB, which enhances inflammation, plays a crucial role in the regulation of pyroptosis [22,23].

Due to their pluripotency and self-renewal capacity, MSCs have become a key research target for clinical therapies, especially for tissue regeneration and the treatment of immune disorders [24]. However, transplantation of MSCs still has many safety concerns, such as tumorigenicity, immunocompatibility, and thrombosis [25,26]. The secretome of ADSCs contains many cytokines, chemokines, and exosomes, which are known to inhibit apoptosis, modulate immune responses, and promote regeneration [27]. Not surprisingly, the ADSC-secretome has demonstrated therapeutic effects against inflammatory and immune-related diseases [28,29,30].

Typically, the efficacy of the MSC secretome mirrors that of its originating cell type, and its composition and content are influenced by the parent cell [31]. Consequently, choosing the appropriate producer cell type for therapeutic purposes is of paramount importance. Due to their abundance, ease of access, and absence of ethical dilemmas, adipose-derived mesenchymal stem cells (ADSCs) hold significant promise in stem cell therapy [32,33]. Our prior research has shown that ADSCs can diminish inflammatory responses induced by HIRI and reduce hepatocyte apoptosis in minipigs [34,35], and Jiao et al. found that ADSC-secretome possessed an equivalent therapeutic effect and acted similarly to ADSCs [36,37]. Nevertheless, there is currently no documentation regarding the influence of ADSC-secretome on cellular pyroptosis triggered by liver injury in minipigs. Furthermore, the possible therapeutic effects of both ADSCs and their secretome in relation to pyroptosis amid liver injury in miniature pigs remain unexplored.

## 2. Materials and Methods

### 2.1. Animals

A total of 24 Guangxi Bama miniature pigs, aged between 4 and 6 months and weighing between 20 and 25 kg (with an equal number of males and females), were used. All animal experimentation protocols were approved by the Animal Management Committee of Northeast Agricultural University.

### 2.2. Establishment of HIRI and Hepatectomy Model

**Figure 1:**
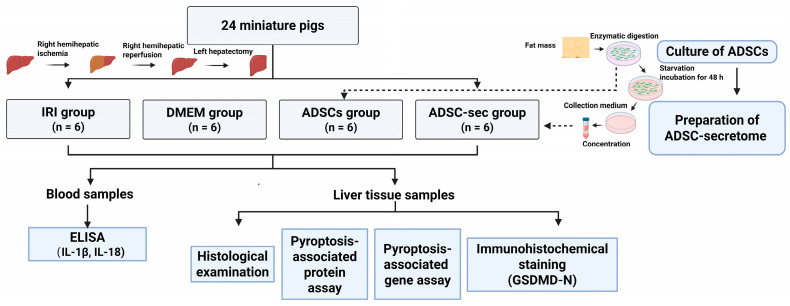
Flow chart of the experiment.

In this study, a model of HIRI in conjunction with left hepatic lobectomy was established in minipigs by inducing ischemia in the liver’s right lobe for a duration of 60 min, followed by a left hemi-hepatectomy [38]. As shown in Figure 1, the animals were categorized into four groups: untreated model (IRI), DMEM (DMEM), ADSCs intervention (ADSCs), and ADSCs secretome intervention (ADSC-sec), and, respectively, injected with 5 mL saline, DMEM, ADSCs suspension (1 × 10^6^ cells/kg) and ADSCs culture supernatant (collected from 1 × 10^6^ cells/kg). All procedures were performed by the same operator. Collection of serum and liver tissue samples occurred prior to surgery, as well as on the 1, 3, and 7 days following the operation.

### 2.3. Culture of ADSCs

Fresh fat mass was taken from the groin of miniature pigs, with fascia and blood vessels excised. The fat mass was diced into approximately 1 mm^3^ fragments and subjected to digestion using 0.1% collagenase type (1:1) in a water bath set to 37 °C for 40 min. Upon completion of the reaction, the tissue homogenate was subjected to filtration and centrifugation, followed by treatment for red blood cell lysis to isolate the cell pellet. Discarding the supernatant allowed for the final re-suspension of the cells in low-sugar DMEM supplemented with 10% FBS. The cells were seeded into culture flasks and cultured at 37 °C with 5% CO_2_. The identification of ADSCs can be found in previous studies [35]. Cells from the fourth passage were used for the experiments.

### 2.4. Preparation of ADSC-Secretome

ADSCs from the fourth passage were cultured till >90% confluent. Following the removal of the medium, the cells underwent washing with PBS, after which serum-free starvation medium was introduced. They were incubated for a duration of 48 h, after which the medium was collected and centrifuged to eliminate dead cells and cellular debris. The supernatant underwent filtration with a 0.22 µm sterile filter, and the resulting filtrate was concentrated 24 times using a 3 kDa ultrafiltration concentrator tube (5000 g/min for 1 h). The concentrated ADSC-secretome was subsequently stored at −80 °C.

### 2.5. ELISA

In this study, the levels of interleukin-18 (IL-18) and interleukin-1 beta (IL-1β) present in serum samples were quantified utilizing specific ELISA kits (Jiangsu Jingmei Biotechnology, Yancheng, China), in accordance with the provided instructions.

### 2.6. Histological Examination

Liver tissue samples were carefully preserved in a solution of 4% paraformaldehyde, followed by embedding in paraffin to maintain the integrity of the samples. Once the samples were prepared, they were sectioned into thin slices and stained with hematoxylin and eosin, which facilitated detailed observation under a light microscope. The extent of liver damage was assessed using the Suzuki scoring system. Tissues with no congestion, vacuolization, or necrosis were scored as 0, and those with Serious injuries >60% necrosis were scored as 4 [39].

### 2.7. Western Blotting

Liver tissue samples were added to RIPA protein lysate (Beyotime Biotechnology, Shanghai, China) for grinding, lysis, and centrifugation to isolate total proteins, followed by protein concentration measurement using a BCA kit (Beyotime Biotechnology, Shanghai, China). All protein samples were adjusted to a concentration of 3 μg/μL and subjected to boiling for denaturation. They were then separated using SDS-PAGE, a common technique for distinguishing proteins based on their size. After the separation process, the proteins were transferred to nitrocellulose filter (NC) membranes (Millipore, MA, USA). To prevent non-specific binding during the subsequent incubation steps, the membranes were subjected to a blocking procedure using a 5% skimmed milk solution. Following this, the membranes were incubated overnight with primary antibodies specific to β-actin (1:1000, Cell Signaling Technology, MA, USA), α-tubulin (1:10,000, Proteintech, IL, USA), IL-1β (1:500, ABmart, Shanghai, China), IL-18, caspase1, NLRP3, HMGB1, TLR4 (1:1000, WanLei Biologicals, Shenyang, China), apoptosis-associated speck-like protein containing a CARD (ASC), GSDMD-N (1:1000, Affinity Biosciences, FLA, USA), NF-κBp65 (1:2000, Proteintech, IL, USA), p-IκB (1:1000, Immunoway, TX, USA), p-NF-κBp65 (1:500, Santa Cruz Biotechnology, CA, USA), and IκB (1:1000, Immunoway, TX, USA). The next day, membranes were probed with secondary antibodies (1:10,000, Absin, Shanghai, China). Visualization of positive bands was achieved through the application of an enhanced ECL reagent (Meilunbio, Dalian, China) utilizing the Tanon 5200 (Tanon, Shanghai, China), and quantified using ImageJ 1.53 software.

### 2.8. qRT-PCR

RNA was extracted from liver tissue utilizing the Total RNA Kit (TIANGEN, Beijing, China) following the protocols set forth by the manufacturer and subsequently stored at −80 °C. The extracted total RNA underwent reverse transcription to cDNA with the aid of the PrimeScript™ RT Kit (Takara, Tokyo, Japan). The RT-qPCR was conducted on the Light Cycler 480 (Roche, Basel, Switzerland). The expression levels of the target genes were measured using the 2^−ΔΔCT^ method. The primer sequences used in this study can be found in [40].

### 2.9. Immunohistochemical Staining

Liver tissues underwent fixation in a tissue fixative, subsequently embedded in paraffin, and sectioned. Following a deparaffinization process utilizing xylene, the tissue sections received treatment with 3% H_2_O_2_ to inhibit endogenous peroxidase activity, and then heat treatment in sodium citrate buffer was performed for antigen retrieval. To further improve the specificity of the staining, any non-specific binding was blocked with BSA. After this blocking step, the sections were incubated with primary antibodies specific to the target antigens, followed by secondary antibodies designed to bind to the primary antibodies, thereby amplifying the signal. The development of the antibody reaction was achieved using DAB. Subsequently, the tissue sections were dehydrated to remove any remaining water, sealed with a resin to preserve the samples, and ultimately examined under a light microscope for analysis.

### 2.10. Statistical Analysis

The analysis of data was conducted with SPSS version 18.0, and findings were represented as mean ± standard deviation. A one-way analysis of variance (ANOVA) was employed for comparisons between groups, with a *p*-value < 0.05 considered to be statistically significant.

## 3. Results

### 3.1. ADSC-Secretome Alleviated HIRI in a Minipig Model

**Figure 2:**
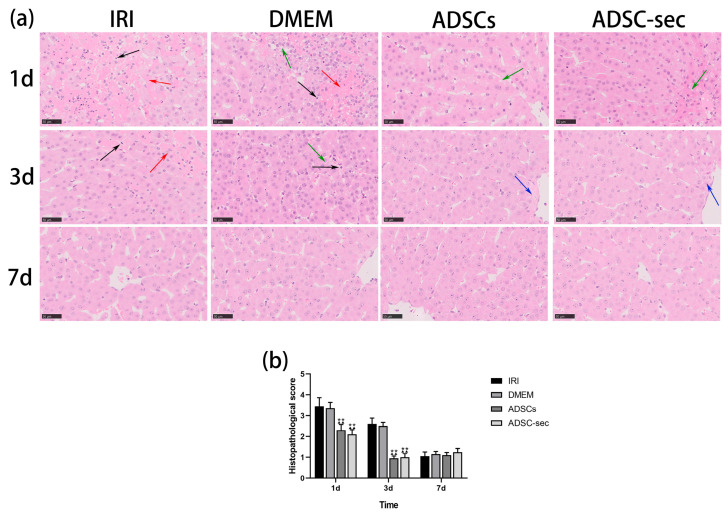
ADSC-secretome alleviated HIRI. (**a**) H&E staining of liver tissue at 1 d, 3 d, and 7 d postoperatively in each group. A total of five distinct fields were chosen for every sample analyzed. The green arrows illustrate the vacuolar degeneration of hepatocytes, while the red arrow indicates the presence of hemorrhage. The blue arrows depict the swelling of hepatocytes, and the black arrows highlight the infiltration of inflammatory cells (scale: 100 μm). (**b**) Presented are Suzuki scores for each group. ^▲▲^ *p* < 0.01 compared to the IRI group. ** *p* < 0.01 compared to the DMEM group.

As shown in the HE results (Figure 2), the IRI and DMEM groups displayed severe hemorrhaging, along with significant vacuolization and infiltration of numerous inflammatory cells on postoperative days 1 and 3, and their Suzuki scores were similar (*p* > 0.05). The Suzuki scores for the two intervention groups were comparable (*p* > 0.05). One week following the surgery, the extent of liver tissue damage observed in the small pigs was minimal, and no substantial differences in the Suzuki scores were noted between the groups (*p* > 0.05).

### 3.2. ADSC-Secretome Attenuated HIRI-Induced Inflammation

**Figure 3:**
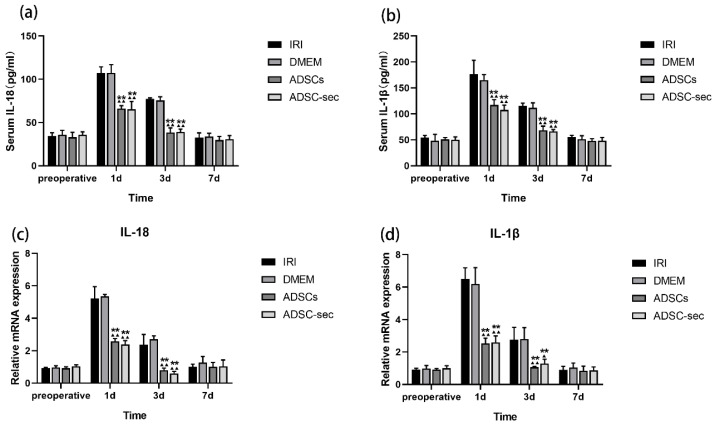
ADSC-secretome attenuated HIRI-induced inflammation. (**a**,**b**) The concentrations of IL-18 and IL-1β in serum were measured in various groups at preoperative, as well as 1 day, 3 days, and 7 days postoperatively. (**c**,**d**) The mRNA levels of *IL-1β* and *IL-18* were evaluated across different groups. Three replicates were performed for each sample. The relative expression of mRNA was standardized against the corresponding levels of β-actin. ^▲^ *p* < 0.05 and ^▲▲^ *p* < 0.01, compared to the IRI group. ** *p* < 0.01, compared to the DMEM group.

Copious amounts of inflammatory cytokines are released from the liver during HIRI. Studies show that pyroptosis facilitates the maturation and secretion of IL-18 and IL-1β [41]. Preoperative cytokine concentrations were comparable across the experimental groups (*p* > 0.05). Serum concentrations of IL-18 and IL-1β in two control groups showed a similar increase on the first and third days following HIRI (*p* > 0.05; Figure 3a,b). Postoperative days 1 and 3 saw significant reductions in concentrations of IL-18 and IL-1β in both the ADSC-sec group and the ADSCs group when compared to the IRI group and the DMEM group (*p* < 0.01), with no notable differences between the two intervention groups (*p* > 0.05). On the seventh day after surgery, no notable discrepancies were found among the groups (*p* > 0.05). Consistent with the serum findings, both the ADSCs and ADSC-secretome groups demonstrated a considerable reduction in mRNA expressions of *IL-18* and *IL-1β* within liver tissue on the first and third postoperative days (*p* < 0.01), again showing similar results between the groups (*p* > 0.05; Figure 3c,d). The results indicate that both ADSC-secretome and ADSCs are effective in suppressing the inflammatory response triggered by HIRI, exhibiting comparable effects.

### 3.3. ADSC-Secretome Inhibited HIRI-Induced NLRP3 Inflammasome Activation

**Figure 4:**
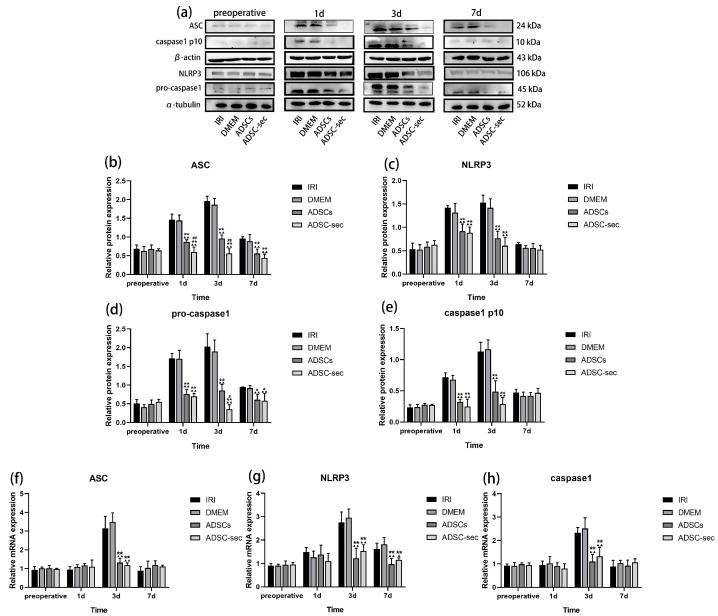
ADSC-secretome inhibited HIRI-induced NLRP3 inflammasome activation. (**a**) Representative Western blot results for ASC, NLRP3, pro-caspase1, and caspase1p10. (**b**–**e**) Evaluation of protein expression levels for ASC, NLRP3, pro-caspase1, and caspase1p10. The expression levels of ASC and caspase1p10 proteins were adjusted to β-actin levels, while NLRP3 and pro-caspase1 expression levels were normalized based on α-tubulin. (**f**–**h**) Assessment of gene expression levels for *ASC*, *NLRP3*, and *caspase1*. Three replicates were performed for each sample. The relative mRNA expression was normalized to the levels of β-actin. ^▲^ *p* < 0.05 and ^▲▲^ *p* < 0.01, compared to the IRI group. * *p* < 0.01 and ** *p* < 0.01, against the DMEM group. ^#^ *p* < 0.05 and ^##^ *p* < 0.01, relative to the ADSCs group.

The NLRP3 inflammasome is essential for mediating inflammation and has been recognized as a promising novel target for anti-inflammatory interventions [42]. According to Figure 4a–e, before surgery, the levels of various proteins were similar in all experimental groups (*p* > 0.05). On days 1 and 3 following HIRI, the IRI and DMEM groups exhibited an increase in the levels of inflammasome-related proteins, with expression levels in both groups being comparable (*p* > 0.05). Conversely, both intervention groups exhibited a marked reduction in the expression of these proteins at identical time points (*p* < 0.05). While the ADSC-sec group showed a greater reduction in the inflammasome-related proteins compared to the ADSCs group, only ASC and pro-caspase1 showed significant differences (*p* < 0.05). The mRNA level assay results (Figure 4f–h) showed that the expression levels of various mRNAs were comparable among the preoperative groups (*p* > 0.05). On the first and third days post-operation, transcript levels of *ASC*, *NLRP3*, and *caspase1* did not show significant variations between the IRI and DMEM groups (*p* > 0.05). However, notable reductions were observed in the ADSCs and ADSC-secretome groups by day 3 post-operation (*p* < 0.05). By the seventh day following surgery, *NLRP3* mRNA levels remained significantly reduced in both the ADSCs and ADSC-secretome groups compared to the IRI and DMEM groups (*p* < 0.05), whereas other indices remained comparable among the groups (*p* > 0.05). These findings indicated that both ADSC-secretome and ADSCs can block NLRP3 inflammasome activation, and the inhibitory effect of ADSC-secretome may be superior.

### 3.4. ADSC-Secretome Reduced GSDMD Expression in the Liver Tissues After IRI

**Figure 5:**
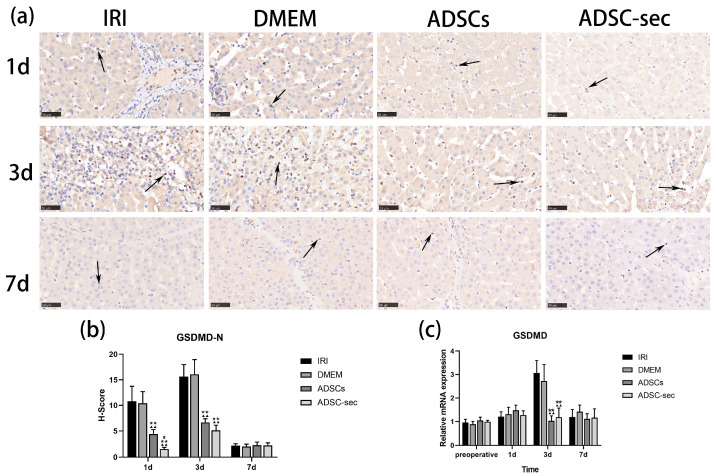
ADSC-secretome reduced GSDMD expression in the liver tissues after IRI. (**a**) Immunohistochemical staining of the GSDMD protein. The immune cells are represented by the black arrows. The H-Scores in five random fields from each group were determined using ImageJ 1.53 software. (**b**) Results of GSDMD immunohistochemical analysis. (**c**) Analysis of *GSDMD* gene expression levels. Three replicates were performed for each sample. The relative levels of mRNA expression were normalized against the corresponding levels of β-actin. ^▲▲^ *p* < 0.01, compared to the IRI group. ** *p* < 0.01, compared to the DMEM group. ^#^ *p* < 0.05, compared to the ADSCs group.

GSDMD is a synergistic effector of inflammasomes and participates in the typical inflammasome-induced pyroptosis cascade. Upon the occurrence of pyroptosis, GSDMD undergoes cleavage, resulting in the release of GSDMD-N, which possesses intrinsic pore-forming capabilities. The GSDMD-N fragment interacts with membrane lipids, creating pores that facilitate the release of cytokines and various cytoplasmic components [43]. Immunohistochemical analyses indicated positive staining within immune cells infiltrating the liver. Immunostaining of the liver tissues was similar in the expression of GSDMD protein between the control groups on days 1 and 3 post-operation (*p* > 0.05; Figure 5a,b). In comparison to the control group, both intervention groups significantly reduced GSDMD-N protein expression on the first and third postoperative days (*p* < 0.01), and the ADSC-secretome group had a relatively stronger inhibitory effect on day 1 (*p* < 0.05) on the first postoperative day. At 7 days postoperatively, GSDMD-N expression levels were similar in the four groups (*p* > 0.05). Furthermore, *GSDMD* mRNA levels were considerably lower in both intervention groups when contrasted with the IRI and DMEM groups (*p* < 0.01; Figure 5c) 3 days after HIRI, although no noteworthy differences were observed at other time intervals (*p* > 0.05). Collectively, these findings imply that both the ADSC-secretome and ADSCs may impede pyroptosis mediated by the NLRP3 inflammasome in the liver by inhibiting GSDMD cleavage.

### 3.5. ADSC-Secretome Inhibited the HMGB1/TLR4/NF-κB Pathway During HIRI

**Figure 6:**
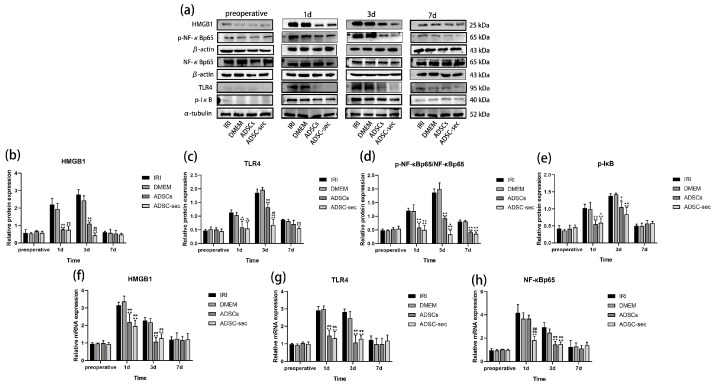
ADSC-secretome inhibited the HMGB1/TLR4/NF-κB signaling pathway during HIRI. (**a**) Representative Western blot analysis of HMGB1, TLR4, p-NF-κBp65/NF-κBp65, and p-IκB. (**b**–**e**) The levels of HMGB1, TLR4, p-NF-κBp65/NF-κBp65, and p-IκB proteins were evaluated. The protein levels of HMGB1, p-NF-κBp65, and NF-κBp65 were adjusted against the respective levels of β-actin. For TLR4 and p-IκB, their protein expression levels were normalized to α-tubulin levels. (**f**–**h**) The gene expression analysis for *HMGB1*, *TLR4*, and *NF-κBp65* was conducted. Three replicates were performed for each sample. The relative mRNA levels were standardized with reference to β-actin. The significance of results is indicated as ^▲^ *p* < 0.05, ^▲▲^ *p* < 0.01, when compared to the IRI group; * *p* < 0.01 and ** *p* < 0.01, relative to the DMEM group; ^#^ *p* < 0.05 and ^##^ *p* < 0.01, in comparison to the ADSCs group.

HMGB1 interacts with TLR4, thereby activating the NF-κB signaling pathway, an essential component for NLRP3 inflammasome activation [23]. As illustrated in Figure 6a–e, preoperative levels of proteins linked to the HMGB1/TLR4/NF-κB pathway remained similar in all groups (*p* > 0.05). On the first and third days following surgery, the levels of HMGB1, TLR4, p-NF-κBp65/NF-κBp65, and p-IκB proteins rose in both control groups, while they were notably lower in both treatment groups (*p* < 0.05). The secretome from ADSCs demonstrated a more pronounced downregulation of these proteins when compared to the ADSCs alone; however, significant differences in expression were only observed for HMGB1, TLR4, and p-NF-κBp65/NF-κBp65 between the ADSC-sec group and the ADSCs group (*p* < 0.05). Figure 6f–h shows no significant differences in mRNA expression levels among the preoperative groups. The transcript levels of *HMGB1*, *TLR4*, and *NF-κBp65* exhibited similar patterns in the IRI and DMEM groups on the first and third days following surgery (*p* > 0.05). A notable decrease in the mRNAs of *HMGB1* and *TLR4* was observed in the two treatment groups in comparison to the two control groups (*p* < 0.01), with *NF-κB* mRNA showing downregulation in the ADSC-secretome group (*p* < 0.01) one day post-HIRI. Compared to the IRI group and the DMEM group, both intervention groups led to a substantial reduction in the gene expression levels of all three genes at postoperative days 3 (*p* < 0.01). These results indicate that both ADSC-secretome and ADSCs have the ability to inhibit the HMGB1/TLR4/NF-κB pathway. In summary, we can deduce that the inhibitory action of ADSC-secretome on HIRI-induced pyroptosis may be facilitated through the suppression of the HMGB1/TLR4/NF-κB signaling cascade.

## 4. Discussion

HIRI represents a critical concern during liver resections and transplant procedures, adversely affecting postoperative liver function recovery. There has been significant interest in deciphering the molecular mechanisms of HIRI, aiming to discover new therapeutic targets [44,45]. Pyroptosis, a form of programmed cell death characterized by inflammation [46], occurs during HIRI following activation of the NLRP3 inflammasomes. The copious amounts of pro-inflammatory factors released as a result further aggravate liver injury [47]. Several studies have shown that inhibiting the pyroptosis cascade can effectively attenuate HIRI [48,49,50].

ADSCs have multipotent differentiation ability and exert paracrine effects through their secreted factors [51]. ADSC-secretome has demonstrated good therapeutic effects against IRI-induced organ damage [52]. One particular investigation revealed that the ADSC-secretome markedly lowered serum levels of ALT, AST, and ALP—parameters that signify improved liver function—while also facilitating liver regeneration post-IRI in conjunction with partial hepatectomy in minipigs [37]. In our study as well, the ADSC-secretome alleviated liver injury caused by HIRI.

NLRP3 functions as an intracellular pattern-recognition receptor that either directly recruits precursor forms of caspase1 or utilizes ASC, leading to the assembly of protein complexes referred to as inflammasomes [53]. Upon activation, these inflammasomes trigger caspase1, facilitating the maturation and subsequent release of cytokines IL-1β and IL-18 [54]. Numerous investigations have indicated that MSCs and MSC-derived secretome can mitigate inflammation by thwarting the production of the NLRP3 inflammasome. For instance, a study by Li and colleagues showed that ADSCs diminished IL-1β secretion by restricting mitochondrial ROS production, which further obstructed the NLRP3 inflammasome and alleviated inflammation linked to acne [55]. In another investigation, it was found that MSC-secretome lessened inflammatory responses induced by LPS in human gingival epithelial cells by impeding NLRP3 inflammasome activation [56]. Previous studies have demonstrated that the NLRP3 inflammasome is involved in the progression of HIRI [57]. We observed that the secretome from ADSCs considerably lowered the levels of NLRP3, ASC, and caspase1 in liver tissues, reduced the secretion of IL-18 and IL-1β, and hindered NLRP3 inflammasome activation. The activated form of caspase1 acts on the GSDMD protein, leading to the release of its GSDMD-N, which subsequently forms pores in the cellular membrane [58], instigating pyroptosis. In a hepatic resection injury model, a deficiency in GSDMD was shown to prevent the activation of hepatic regeneration termination factors such as activin A and acetylheparin sulfate proteoglycan 3, which consequently enhanced liver regeneration [59]. Our study’s findings indicated that the ADSC-secretome led to a reduction in GSDMD expression in hepatic tissues and inhibited the process of pyroptosis. In conjunction with our earlier discovery that ADSC-secretome facilitates liver regeneration [37], we propose that this may be associated with the inhibitory influence of ADSC-secretome on GSDMD. Additional research is still required to further investigate this hypothesis.

HMGB1 is critical in the progression of liver injuries and related diseases, being implicated in several conditions, including NAFLD, hepatocellular carcinoma, and HIRI [60]. This protein functions as a damage-associated molecular pattern that triggers innate inflammatory responses by attaching to the surface receptor TLR4 [61]. The stimulation of the HMGB1/TLR4 signaling pathway has been shown to stimulate the NLRP3 inflammasome [62]. In this context, research by Ding et al. revealed that panaxynol could impede the synthesis of the NLRP3 inflammasome by influencing the HMGB1/TLR4/NF-κB pathway, thus reducing cellular sepsis caused by ischemia–reperfusion injury in the heart following automotive incidents [23]. Related investigations into HIRI have demonstrated that a specific drug can decrease cellular pyroptosis induced by the NLRP3 inflammasome by inhibiting the TLR4/NF-κB pathway [63,64]. In line with our results, we observed that the ADSC-secretome reduced the levels of HMGB1, TLR4, and NF-κB. Therefore, the ADSC-secretome may mitigate cellular pyroptosis induced by hepatic IRI through suppressing the HMGB1/TLR4/NF-κB signaling pathway.

The MSC-secretome represents a potentially valuable pharmaceutical agent that comprises proteins, genetic components, and vesicles capable of being transferred to various cells, thereby exerting therapeutic effects [65]. At present, the MSC-secretome has attracted considerable interest within the field of regenerative medicine, particularly concerning respiratory, neurological, and liver disorders [66]. Furthermore, the MSC-secretome appears to share biological functions with MSCs in the context of autoimmune and immune-mediated inflammatory conditions, while exhibiting comparatively lower immunogenicity [67]. Our findings indicate that the ADSC-secretome demonstrates effects akin to those of ADSCs in the suppression of HIRI-induced cellular pyroptosis. This supports the theoretical foundation for utilizing ADSC-secretome as a substitute for ADSCs.

While our research indicated that the ADSC-secretome suppresses HIRI-induced pyroptosis in minipigs and that it exerts regulatory influence over the HMGB1/TLR4/NF-κB pathway, we have only conducted a preliminary in vivo investigation, which has inherent limitations in our assay methodology. Consequently, we will turn our attention to analyzing the mechanism through which the ADSC-secretome operates.

## 5. Conclusions

ADSC-secretome attenuated HIRI combined with partial hepatectomy in minipigs by inhibiting pyroptosis induced by the NLRP3 inflammasome. Compared with ADSCs, ADSC-secretome may have a better intervention effect, and warrants further investigation as a cell-free alternative for the treatment of liver injury.

## Data Availability

The corresponding author can provide the data that underpins the findings of this study upon request.

## Abbreviations

The following abbreviations are used in this manuscript:

MSCs	Mesenchymal stem cells
ADSC-secretome	Adipose mesenchymal stem cell-secretome
HIRI	Hepatic ischemia–reperfusion injury
PH	Partial hepatectomy
